# Incidence and pattern of technical complications in balloon-guided osteoplasty for depressed tibial plateau fractures: a pilot study in 20 consecutive patients

**DOI:** 10.1186/1754-9493-7-8

**Published:** 2013-03-01

**Authors:** Cyril Mauffrey, Ryan Fader, E Mark Hammerberg, David J Hak, Philip F Stahel

**Affiliations:** 1 Department of Orthopaedic Surgery, Denver Health Medical Center, University of Colorado, School of Medicine, 777 Bannock Street, Denver, CO 80204, USA

## Abstract

**Background:**

Inflation bone tamps are becoming increasingly popular as a reduction tool for depressed tibial plateau fractures. A number of recent publications have addressed the technical aspects of balloon inflation osteoplasty. However, no study has yet been published to describe the technical limitations, intraoperative complications, and surgical bailout strategies for this new technology.

**Methods:**

Observational retrospective study of all patients managed with inflatable bone tamps for depressed tibial plateau fractures between October 1, 2010 and December 1, 2012. The primary outcome parameter was the rate of complications, which were stratified into “minor” and “major” depending on the necessity for altering the surgical plan intraoperatively, and based on the risk for patient harm. This study was approved by the Institutional Review Board of the State of Colorado.

**Results:**

A consecutive series of 20 patients were managed by balloon inflation osteoplasty for depressed tibial plateau fractures during the 15 months study period. The mean age was 42.8 years (range 20–79), with 9 females and 11 males. A total of 13 patients sustained an adverse intraoperative event (65%), with three patients sustaining multiple technical complications. Minor events (*n* = 8) included the burst of a balloon with extrusion of contrast dye, and the unintentional posterior wall displacement during balloon inflation. Major events (*n* = 5) included the intra-articular injection of calcium phosphate in the knee joint, and the inability to elevate the depressed articular fragment with the inflatable bone tamp.

**Conclusion:**

The observed intraoperative complication rate of 65% reflects a steep learning curve for the use of inflation bone tamps to reduce depressed tibial plateau fractures. Specific surgical bailout options are provided in this article, based on our early anecdotal experience in a pilot series of 20 consecutive cases. Patients should be advised on the benefits and risks of this new technology as part of the shared decision-making process during the informed consent.

## Background

Depressed tibial plateau fractures remain technically demanding for orthopaedic trauma surgeons [1-3]. The main challenges consist of achieving anatomic joint reduction in conjunction with stable fracture fixation, to allow early range of motion of the knee, with the aim of achieving good functional outcomes [4]. Further limitations include the risk of a residual intra-articular step-off after insufficient articular reduction [5], and the adequacy of bone grafting options to fill the metaphyseal void after fracture reduction with conventional bone tamps [6-9]. Recent advances in technique include less-invasive approaches in conjunction with stable fixation using a new generation of angular-stable implants [10,11], and the appealing option of arthroscopy-guided fracture reduction and fixation [12,13]. Balloon-guided minimal-invasive fracture reduction, using an inflatable bone tamp, represents a “cutting edge” emerging technique which has been recently described in numerous publications [14-19]. The advantages of this new technique are intuitive, by *(1)* minimal-invasive percutaneous approaches which preserve the soft tissue envelope and avoid the necessity of a formal open arthrotomy, *(2)* by allowing a controlled gradual elevation of the depressed articular fracture fragment under fluoroscopic or arthroscopic guidance and (3) thanks to a large surface area, by allowing small fragments to be elevated simultaneously [15,16]. A further advantage, as described for the technique of balloon-guided kyphoplasty for vertebral fractures, consists in the creation of a cancellous bone void which allows a safe distribution of fluid-phase bone cement without pressure-induced leakage into the surrounding tissues [20]. The use of fast-setting calcium phosphate appears to represent a safe and efficient technique for filling the metaphyseal void and providing adequate support for the articular surface to prevent secondary subsidence [14,16]. However, as with any appealing new surgical technique, the early enthusiasm regarding potential benefits must be critically weighed against potential complications and the risk of inducing patient harm on the surgical “learning curve” until a new technique is well established and validated [21]. To date, the available evidence supporting the concept of balloon-guided reduction of tibial plateau fracture is restricted to cadaveric studies, anecdotal case reports, and “expert opinion” technical notes [14-18]. Indeed, we have yet to demonstrate the supremacy of this new minimal-invasive technique, compared to the use of traditional bone tamps in “standard” open approaches, in prospective, controlled trials.

The present study was designed to analyze and report the rate and pattern of technical complications in a pilot series of 20 consecutive patients treated by inflation osteoplasty for depressed tibial plateau fractures at an academic level 1 trauma center.

## Methods

This study was designed as a retrospective review of a prospective database of patients undergoing balloon-guided inflation osteoplasty for depressed tibial plateau fractures between October 1, 2010, and December 1, 2012. Indications consisted of partial articular (B-type) depressed lateral or medial tibial plateau fractures amenable to balloon-guided reduction (Schatzker type II, III, IV, AO/OTA type 41-B2 and B3). Exclusion criteria consisted of skeletally immature patients <18 years of age, pure “split” fracture patterns (Schatzker type I, AO/OTA 41-B1), and bicondylar tibial plateau fractures (Schatzker V, AO/OTA 41-C types). All patients were consented for the use of balloon-guided osteoplasty, and understood the potential need for intraoperative conversion to an open procedure in case of technical problems with this new system. The inflatable bone tamp (KyphX® Inflatable Bone Tamp, Kyphon, Sunnyvale, CA) is approved by the U.S. Food and Drug Administration (FDA) for indications related to fracture reduction in the spine, tibia, hand, radius, and calcaneus (2004, #K041454).

**Figure 1:**
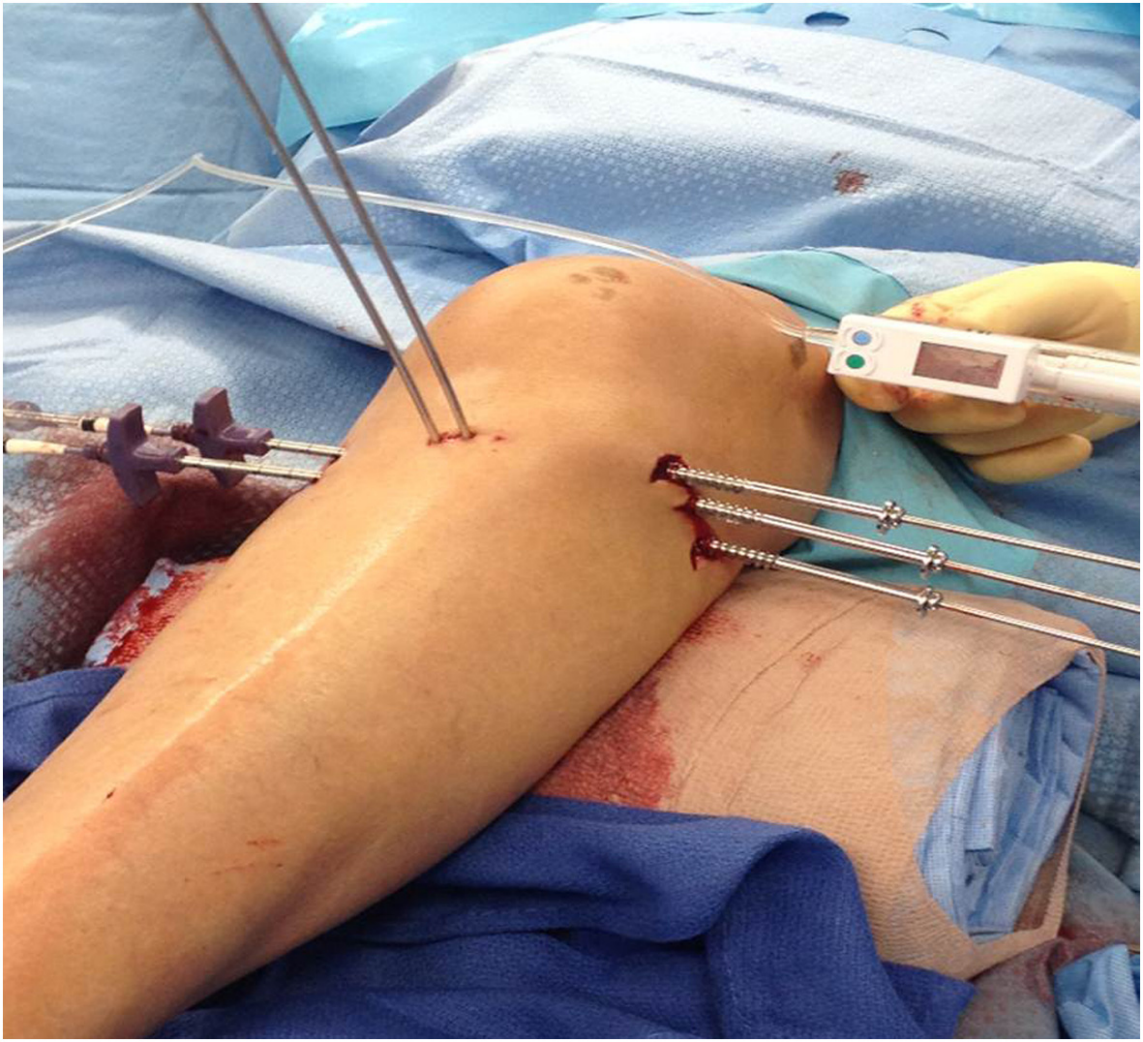
“True” percutaneous technique of balloon-guided inflation osteoplasty with minimal-invasive incisions for medial-to-lateral Jamshidi trocars, antero-posterior rafting wires, and lateral cannulated lag screws.

All surgeries were performed by three fellowship-trained orthopaedic surgeons who are co-authors on this paper (CM: n = 9; PFS: n = 9; DJH: n = 2). The surgical technique has been previously described [16]. Specific technical aspects of the surgical procedure, including the choice of using a buttress plate versus percutaneous cannulated lag screws (Figure 1), as well as the adjunctive use of arthroscopy-guided visualization of the quality of joint surface reduction, were based on individual surgeon’s preference.

Intraoperative technical complications were stratified into “minor” and “major” events, using the following definitions:

(1) “Minor event”: Any unexpected intraoperative event which deviates from the initial surgical plan and from an anticipated uneventful accomplishment of the procedure, but did not mandate a conversion to a different surgical technique, and did not bear the potential for patient harm or an unsatisfactory surgical result.

(2) “Major event”: Any unexpected intraoperative event which resulted in the necessity for conversion to a different surgical technique and/or had the potential for inducing patient harm or an unsatisfactory surgical result.

All adverse events were prospectively entered into an institutional Quality Assurance (QA) database, disclosed to the affected patients per institutional policy [22], and formally peer-reviewed in a standardized fashion at the weekly Departmental QA conference [22]. This study was approved by the Colorado Multiple Institutional Review Board (COMIRB).

## Results

**Figure 2:**
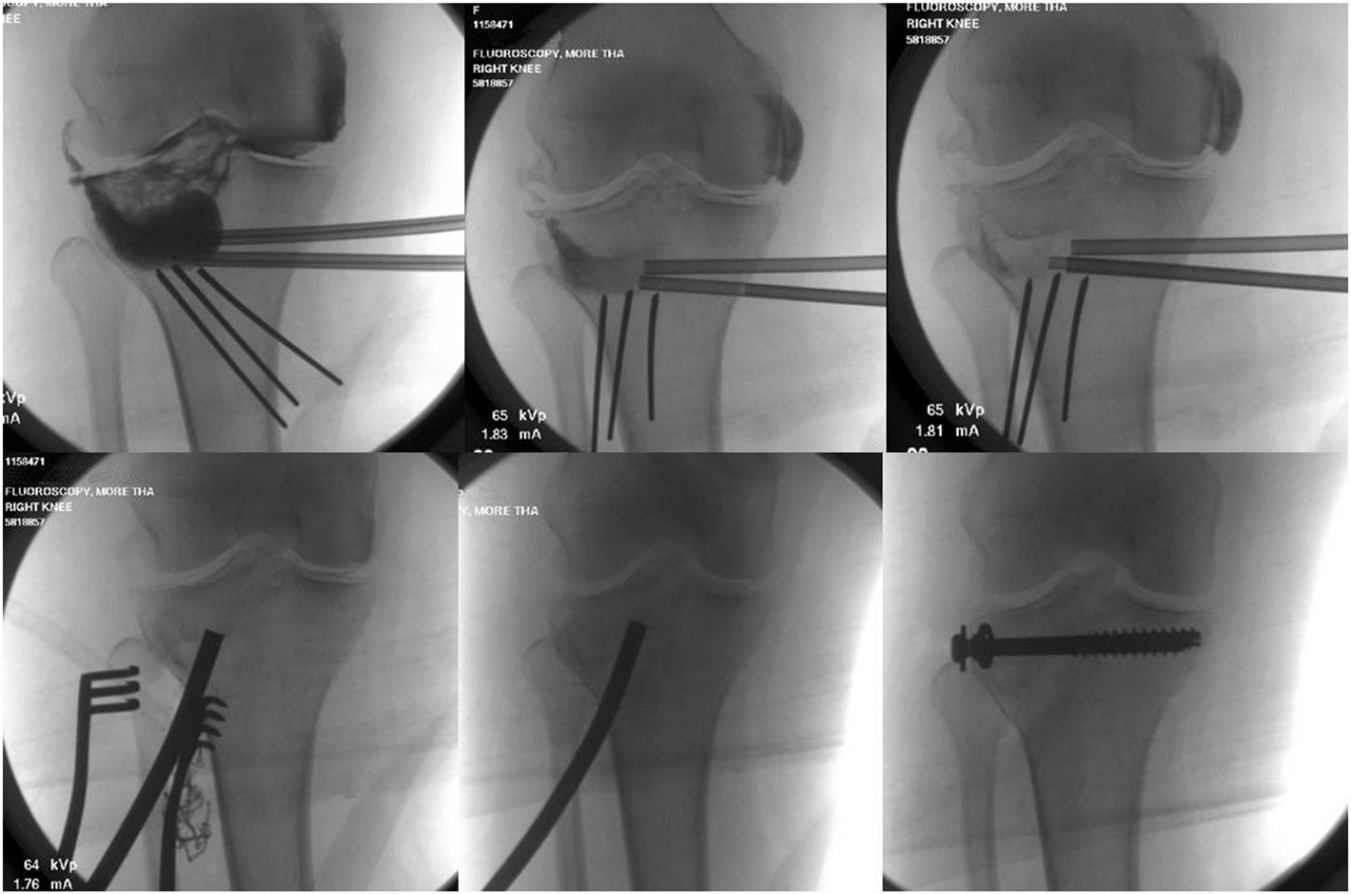
**Intraoperative burst balloon with leakage of the radiographic contrast dye into the knee joint. **This complication is easily resolved by a wash-out with 100-200 cc of sterile saline through the Jamshidi trocar. The bottom panels demonstrate the “bail-out” strategy for the irreducible articular fragment by conversion to the use of a conventional bone tamp and structured bone grafting.

**Figure 3:**
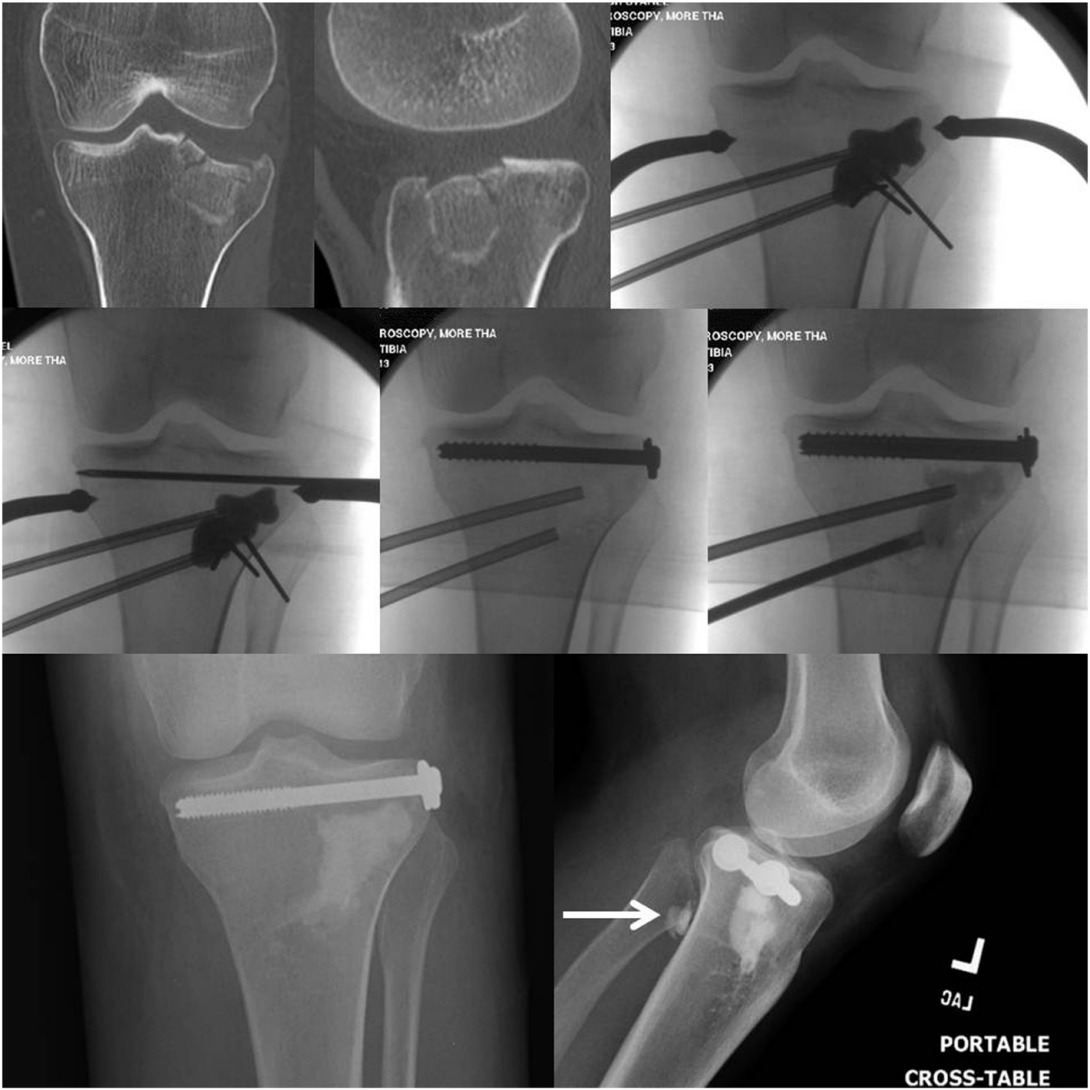
**Intraoperative fluoroscopic views of the “standard” technique of percutaneous balloon-guided inflation osteoplasty, as previously described **[16]**. **The arrow in the bottom right panel points out the small volume of extruded calcium phosphate into the soft tissues, representing a “no harm” event.

**Figure 4:**
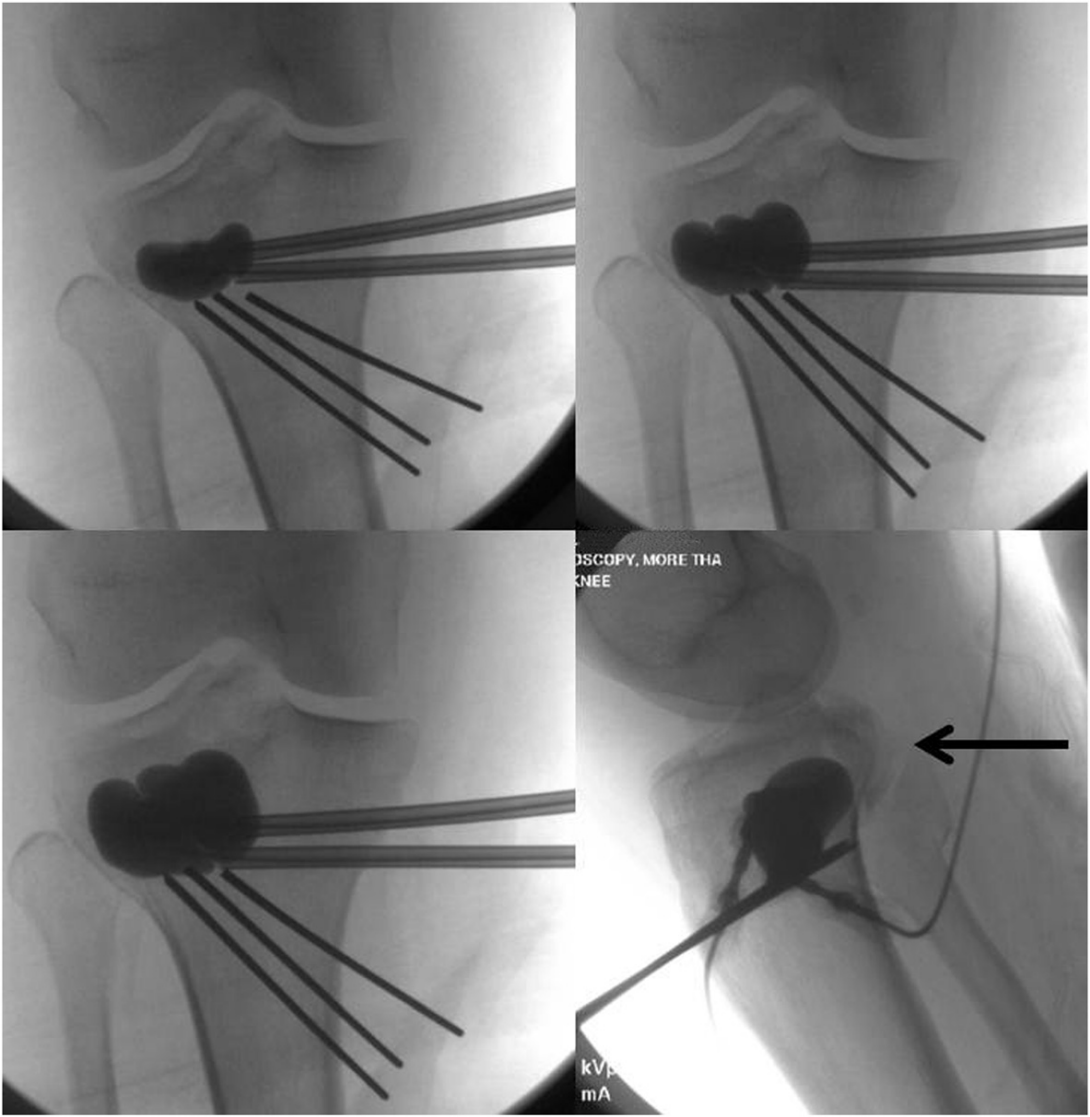
Case example of an irreducible lateral tibial plateau fracture related to the inflated balloon following the “path of least resistance” leading to the displacement of a postero-lateral split fragment (arrow in lower right panel).

Between October 1, 2010, and December 1, 2012, a balloon-guided reduction of depressed tibial plateau fractures using an inflatable bone tamp was applied in a first pilot series of 20 consecutive patients (9 women, 11 men) with a mean age of 42.8 years (range 20–79). Two additional patients were treated by inflation osteoplasty during this time-period but were lost to follow-up and therefore excluded from the study. The fracture classification consisted of Schatzker II (n = 8), Schatzker III (n = 11), and Schatzker IV (n = 1) type injuries. The demographic data of the 20 patients are shown in Table 1. A total of 13 patients (65%) sustained an intraoperative technical complication, of which three patients sustained multiple adverse events. Eight complications were graded as “minor events” based on the definition outlined in the methods section (40%). These minor events consisted of *(a)* the intraoperative burst of a balloon (Figure 2), leading to local leakage of the radiographic contrast dye (*n* = 6); *(b)* the accidental extrusion of calcium phosphate into the posterior soft tissues, which remained asymptomatic (*n* = 1; Figure 3); and *(c)* the intraoperative displacement of the posterior wall fragment of the tibial condyle (*n* = 1; arrow in Figure 4).

**Table 1:** Demographic data of 20 consecutive patients treated by balloon osteoplasty for depressed tibial plateau fractures

**Patient #**	**Age**	**Fx classification (Schatzker)**	**Gender**	**Intraoperative complication**	**Type of fixation**	**Time to surgery (days)**
1	53	II	F	None	Buttress plate	22
2	54	III	M	None	Buttress plate	7
3	31	IV	F	Calcium phosphate extrusion into knee joint	Buttress plate	8
4	50	III	M	None	Buttress plate	9
5	29	II	F	None	Buttress plate	5
6	23	III	F	Calcium phosphate penetration into soft tissues	Perc lag screws	4
7	28	II	F	Balloon burst	Perc lag screws	7
8	44	II	F	Balloon burst	Perc lag screws	2
9	27	II	M	Displacement of posterior wall fragment	Buttress plate	18
10	62	II	M	Balloon burst	Buttress plate	5
11	62	III	M	None	Perc lag screws	3
12	56	III	M	None	Perc lag screws	3
13	38	III	M	Balloon protrusion into knee joint; failed Fx elevation	Buttress plate	16
14	39	III	M	Balloon burst; failed Fx elevation	Perc lag screws	9
15	51	III	F	None	Perc lag screws	8
16	79	III	M	Balloon burst	Perc lag screws	4
17	23	III	F	Balloon burst; failed Fx elevation	Perc lag screws	8
18	56	III	M	Failed Fx elevation	Perc lag screws	25
19	32	II	M	Balloon burst	Perc lag screws	3
20	20	II	F	Balloon burst	Perc lag screws	12

(Fx, fracture; *Perc*, percutaneous).

**Figure 5:**
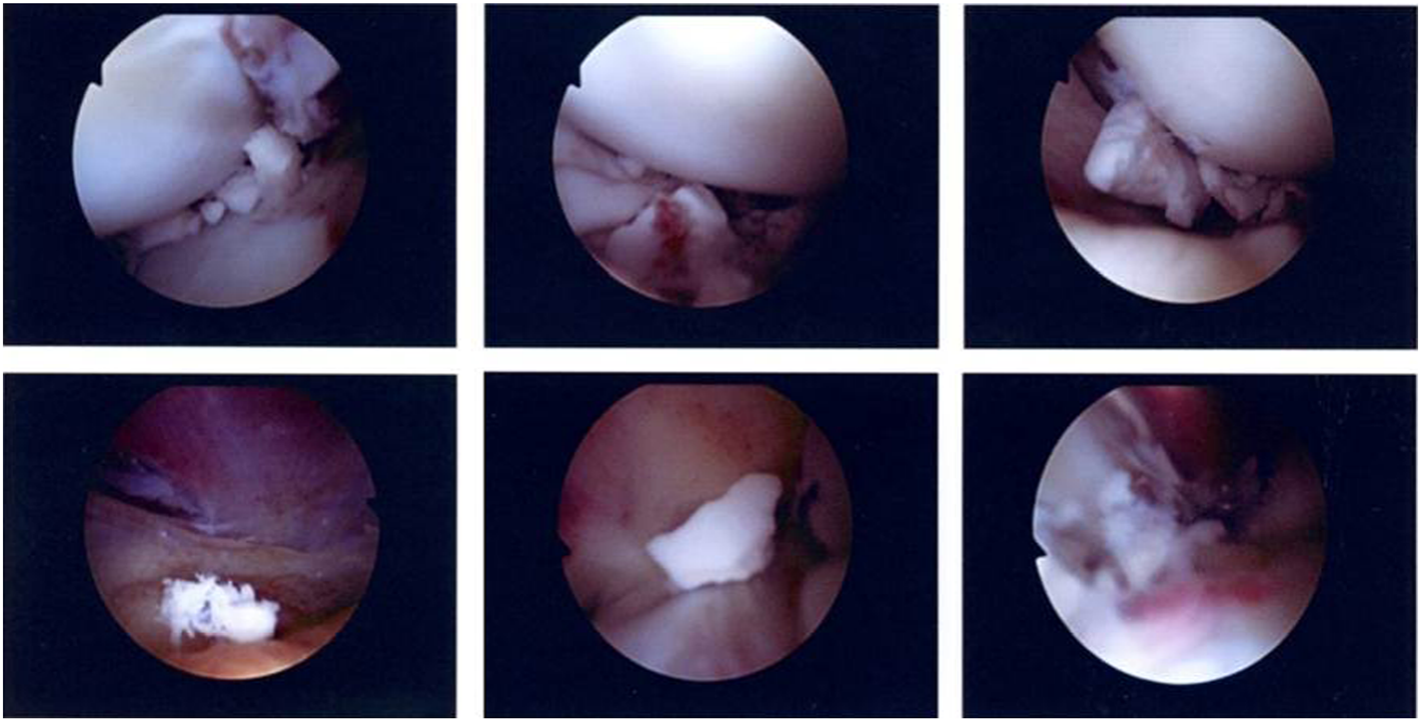
**Arthroscopic images of solidified calcium phosphate crystals within the knee joint after accidental intra-articular extrusion of the fluid-phase bone substitute in a 31-year old patient with a depressed medial tibial plateau fracture. **The patient recovered well from this “major” complication and had an excellent long-term outcome at one year.

**Figure 6:**
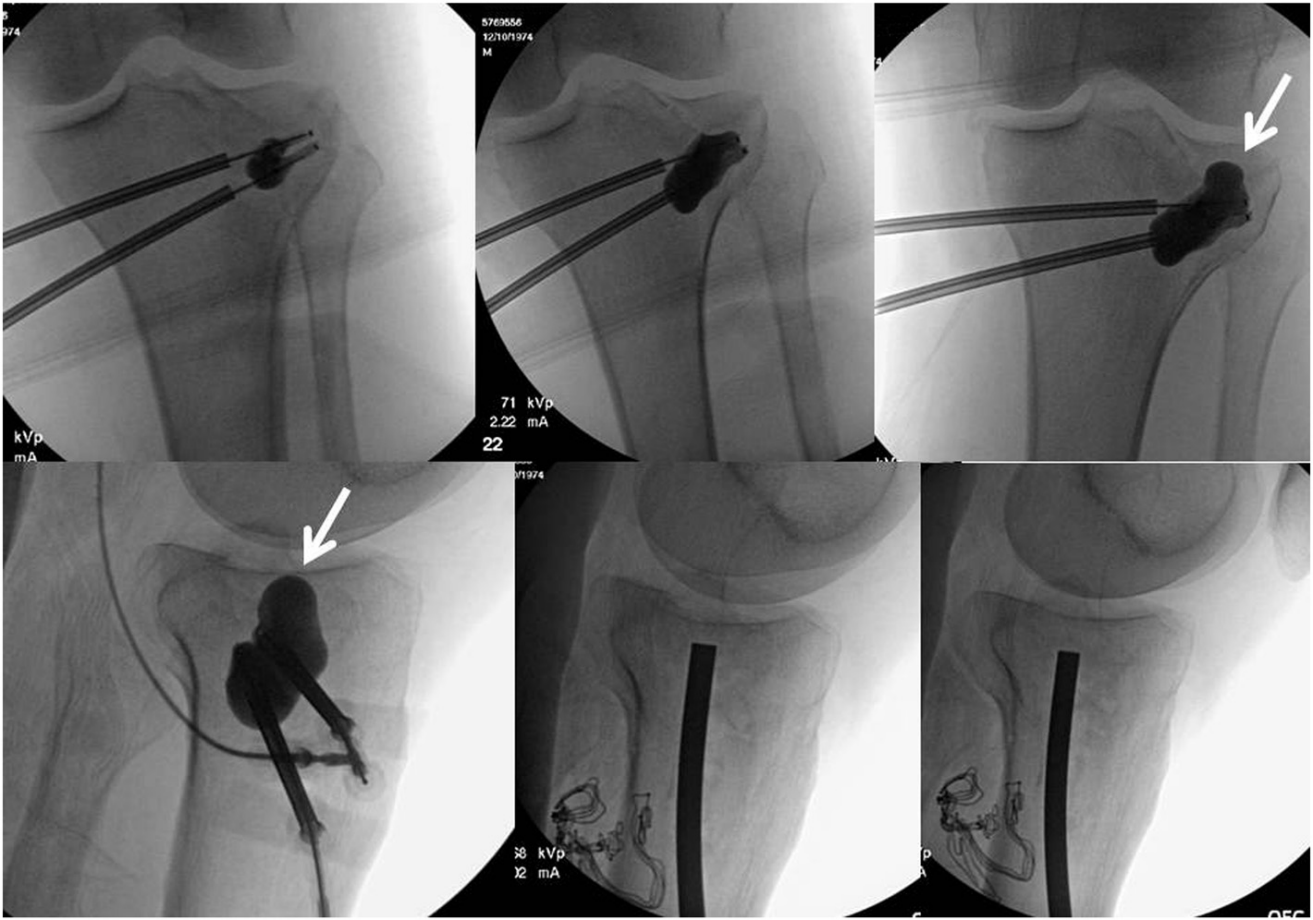
**Intraoperative fluoroscopy films of an irreducible lateral tibial plateau fracture, leading to balloon penetration into the knee joint (arrows). **The salvage strategy consisted of conversion to a “semi-open” technique using a conventional bone tamp and cancellous bone grafting.

“Major events” which altered the course of surgery occurred in 5 patients (25%). These included *(a)* the intra-articular penetration of soluble calcium-phosphate, requiring a formal arthroscopic wash-out and debridement (*n* = 1; Figure 5), and *(b)* the failure to elevate the depressed articular fragment by inflation osteoplasty (Figure 4), which required the conversion to an open procedure using a standard bone tamp (Figure 6) and structured grafting for metaphyseal support (*n* = 4). The most extreme complication pattern attributed to the “non-reducible” group of adverse events consisted of a break-through of the balloon into the knee joint (Figure 6).

**Figure 7:**
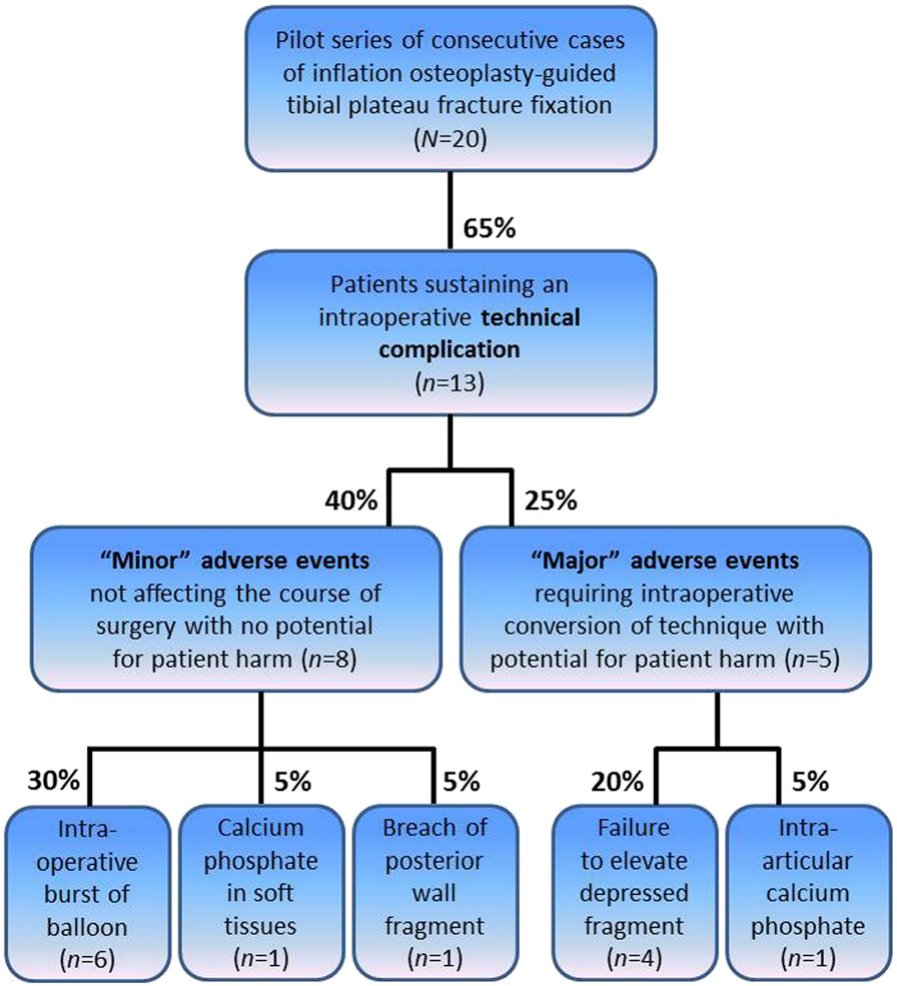
**Incidence and pattern of “major” and “minor” intraoperative complications in a first pilot series of 20 consecutive patients undergoing balloon-guided inflation osteoplasty for depressed tibial plateau fractures. **See text for details and explanations.

The stratification of all patterns of technical complications into “minor” and “major” adverse events is depicted in Figure 7.

## Discussion

This is the first report on the surgical complications related to the emerging technique of balloon-guided osteoplasty for reduction of depressed tibial plateau fractures. Strikingly, we found a complication rate of 65% in our initial series of 20 cases. Most of these complications are fortunately benign and can be adequately managed as “no harm” events by specific bailout strategies, as discussed below. The indications for inflation-guided osteoplasty should be strict. Time since injury, fracture type and comminution around the depressed fragment are aspects that must be considered as part of the surgical decision-making. In our anecdotal experience, we strongly feel that the 2 week mark sets a threshold after which it becomes increasingly difficult to reduce a depressed articular fragment, particularly in young patients. This time-limit is certainly variable and depending on patient age, individual fracture pattern, and energy of the traumatic impact which may lead to impacted and incarcerated articular fragments.

### Standard procedure and technical “tips and tricks”

**Figure 8:**
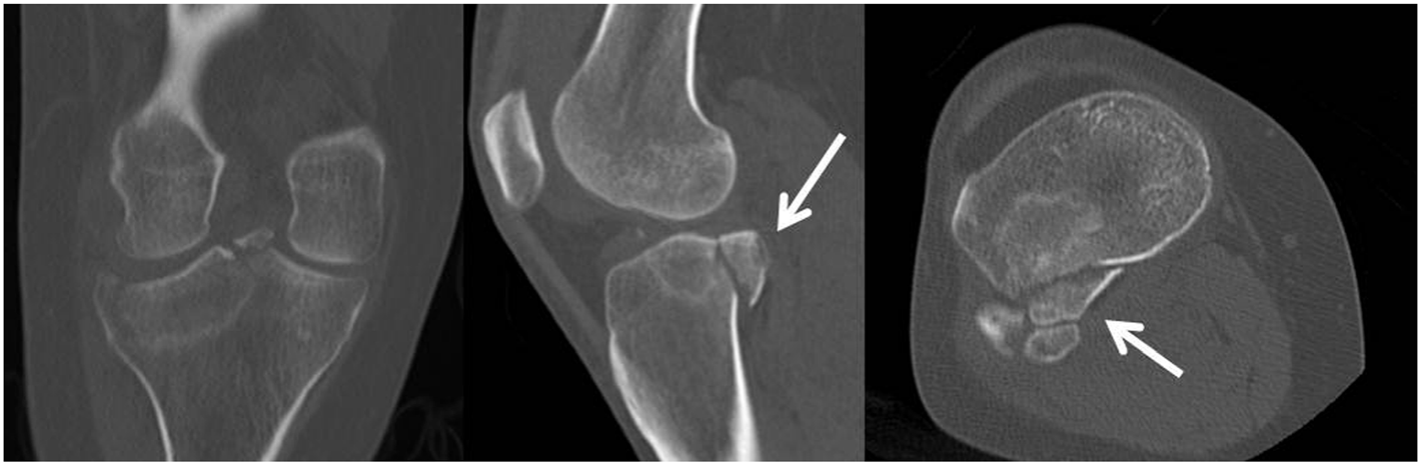
**Based on the adverse experience depicted in Figure **4**, a breach of the posterior wall and presence of a postero-lateral split fragment (arrows) is considered as a contraindication for the use of inflatable bone tamps. **Preoperative radiographs and CT scans must be scrutinized accordingly.

The standard technique used by our group was previously described [16], and is depicted by the case example shown in Figure 3. The type of fracture will also determine which method of reduction to use. In fractures with large splits and an associated comminuted depression, it is often best to perform an open approach and go through the split to visualize the depressed fragment, with or without the need for a formal arthrotomy and submeniscal window. For bicondylar fractures, the energy transfer through the knee is such that a formal surgical approach with a submeniscal window is often needed for anatomic reduction, which precludes from the use of an inflatable bone tamp in most cases. The “ideal” fracture for the use of balloon osteoplasty is one with an isolated depression (Schatzker type III) or a depression associated with a small lateral split (Schatzker type II). Should the split be posterior, in terms of a postero-lateral or postero-medial split fragment (Figure 8), then it is difficult to control reduction as the balloon tends to inflate towards the path of least resistance which will further displace the posterior fragment [3]. We furthermore use arthroscopic guidance in selected cases to directly visualize the reduction and repair or remove the meniscus entrapped in the fracture site, if necessary. We focus on the quality of reduction based on lateral fluoroscopy more than the AP view. However, with the arthroscopy trocar in the lateral compartment and the knee in a “figure-of-four” position renders obtaining a good quality intraoperative AP view of the knee nearly impossible, which is one of the downsides of arthroscopy-assisted reduction.

With regard to the technique of balloon placement, we chose to place the first balloon slightly more distal to the most distal portion of the depressed articular fragment in both the AP and lateral plane under fluoroscopy. This allows us to insert a second cannula just between the first cannula and the point of maximum depression before inflation of the first balloon, and for the more distal balloon to act as a buttress and avoid distal subsidence of the proximal balloon into the weak metaphyseal bone. As an alternative, antero-posterior 2.0 mm or 2.4 mm rafting K-wires (as shown as an example in Figure 1) may replace the need for an adjunctive distal balloon, and avoid distal subsidence into the metaphysis. However, these K-wires have to be placed with diligent fluoroscopic guidance since they may accidentally perforate the balloon, which represents one of the most frequent (“minor”) technical complications seen in this study (Figure 2).

We have recently moved away from using buttress plates for Schatzker type II and III fractures, which require a more extensive soft tissue dissection, and may not be required from a biomechanical standpoint. Instead, we prefer to use percutaneous 6.5 mm or 7.3 mm cannulated screws with washers in most cases, if feasible. This “true” percutaneous technique (Figure 1) preserves the soft tissue envelope in its entirety and likely allows for a faster rehabilitation and biological healing. We recognize that the evidence to support this statement is currently lacking and we encourage new users to get meticulously familiar with the new reduction tool prior to modifying their preferred fixation technique.

The standard rehabilitation concept by our group consists of immediate range of motion of the knee from 0°-90°, with weight-bearing protection of the affected leg for 8–10 weeks postoperatively. Rarely, patients with an unstable knee exam after surgical fracture fixation will be placed in a functional knee brace for 6 weeks.

### “Minor” technical complications (40%)

#### Burst balloon

This was by far the most common intraoperative complication, occurring in 6 patients of 20. There are several hypotheses explaining this phenomenon. The first one is over-inflation of the balloon beyond the failure point. This seems to occur beyond 400 PSI or at a volume superior to 4 cc. The balloon can also fail if it engages into a sharp bony spike, or by accidental perforation during placement of rafting K-wires. Hereby, the balance between the volume of the balloon and its pressure is of crucial importance. If the injury occurred more than 2 weeks prior to surgery, the depressed fragment will start to heal and the pressure developed by the balloon will likely fail before reaching 4 cc of volume, which is below the theoretical failure point of a balloon. The opposite can be true where in presence of osteoporosis, when the tissue pressure surrounding the balloon is so low that the balloon can inflate to a volume of 8 cc without failing. The shape of the balloon on the fluoroscopy can also be an indicator of impending failure. The proximal aspect of the balloon should be concave, accommodating the distal aspect of the depressed articular fragment. Balloons that tend to inflate distally between the rafting wires or laterally will most likely fail unless the balloon and rafting wires are repositioned. The leakage of contrast dye into the knee joint after balloon burst is easily resolved by a wash-out with 100-200 cc of sterile saline through the Jamshidi trocar (Figure 2).

#### Extrusion of calcium phosphate into soft tissues

This is a rare “no harm” event seen in a single patient, and is likely non-preventable. Gradual and diligent injection of calcium phosphate under fluoroscopic guidance will allow to detect a leakage into soft tissues and may require the procedure to be aborted, and replaced by conversion to solid-phase bone substitutes through a small bone window. The “key” aspect of this complication is to avoid protrusion of calcium phosphate into the knee joint under any circumstance, or to recognize and abort immediately, once this “major” complication occurs (see below). This is a complication that can occur using either the novel reduction technique as well as traditional methods of fracture reduction.

#### Displacement of posterior wall

As a basic concept, the inflated balloon will always follow the path of least resistance. Accidental displacement of the lateral split fracture can easily be prevented by placing a loosely tightened pointed reduction clamp to hold the split reduced. The situation is more difficult to control when the split is located postero-laterally, or postero-medially (arrows in Figure 8). In our group’s experience, a posterior wall breach represent a contraindication to the use of the balloon inflation technique, and standard reduction techniques with conventional bone tamps should represent the standard of care for such fracture patterns [3].

### “Major” technical complications (25%)

#### Failure to elevate the depressed articular fragment

In four patients, the balloon failed to elevate the depressed fragment, requiring conversion to a “semi-open” technique of reduction with conventional bone tamps. When reviewing the root cause of these events, the four patients were at 16, 9, 8, and 25 days mark since their injury respectively. The classical picture of failure to elevate the fragment is one where the balloon tends to wrap distally around the rafting wires rather than inflating proximally. The “trapdoor effect” should also be kept in mind. This was described recently in a technical paper which highlighted the importance of not over-compressing the split component of the fracture during the reduction maneuver as this can potentially reduce the space for the depressed articular fragment to be elevated [23].

#### Intra-articular penetration of calcium phosphate

As described for the “minor” complication of calcium phosphate leakage into soft tissues, the injection of the fluid-phase compound must be monitored in a meticulous fashion under fluoroscopic control in both planes, in order to strictly avoid calcium phosphate penetration into the knee joint. This “major” complication occurred in one patient, likely due to poor intraoperative visualization by fluoroscopy, requiring a formal arthroscopic wash-out of the crystallized bone substitute (Figure 5). The patient recovered well from this preventable complication, and had an excellent long-term outcome at one year after surgery.

## Conclusion

In this article, we present a first pilot series of 20 consecutive patients treated by balloon-guided inflation osteoplasty for depressed tibial plateau fractures. Strikingly, the rate of technical intraoperative complications associated to the use of this novel technology was as high as 65%, implying a steep learning curve. None of the complications reported in this study led to any patient harm or adverse outcome. Patients must be counseled in detail about all risks and potential benefits of this new technology, including the high risk of technical intraoperative complications, and the option of a traditional surgical technique must be offered by the surgeon as part of the shared decision-making process.

## Competing interests

Dr. Stahel’s spouse was a salaried employee with Medtronic Spine until April 1, 2012, which coincides with part of the study period of patient enrollment. Dr. Stahel did not receive any monetary or non-monetary compensation, grant support, or any other financial incentives through Kyphon or Medtronic, related to the present study.

## Authors’ contributions

CM and PFS designed this study. CM and RF drafted the first version of the manuscript. CM, PFS, and DJH performed the surgical procedures in the 20 patients included in this study. PFS performed critical revisions to the final version of the manuscript. All authors read and approved the manuscript prior to submission.
